# Preparation of Ester-Crosslinked PI Membranes with Enhanced Gas Selectivity and Plasticization Resistance

**DOI:** 10.3390/membranes16010047

**Published:** 2026-01-20

**Authors:** Yu Li, Jiangzhou Luo, Honglei Ling, Song Xue

**Affiliations:** Tianjin Key Laboratory of Organic Solar Cells and Photochemical Conversion, School of Chemistry & Chemical Engineering, Tianjin University of Technology, Tianjin 300384, China; 16611612022@163.com (Y.L.);

**Keywords:** transesterification reactions, polyimide membranes, gas separation performance, plasticization resistance

## Abstract

Fabricating polyimide (PI) membranes with outstanding anti-plasticization ability and gas separation performance remains a challenge. In this study, two novel diamine monomers, DAMBO (methyl 3,5-diamino-4-methylbenzoate) and DAPGBO (3-hydroxypropyl 3,5-diamino-4-methylbenzoate), were synthesized through esterification reactions. Then, we copolymerized each of these two new monomers with 4,4′-diaminodiphenylmethane (DAM) and 4,4′-(Hexafluoroisopropylidene) diphthalic anhydride (6FDA) separately to yield two monoesterified PIs. Following this, we further prepared the ester-crosslinked PIs by inducing a transesterification crosslinking reaction within the PI-PGBO membrane via thermal treatment. As expected, we found that the formation of cross-linked structures can effectively regulate the microporous structure, enhance its sieving performance, and thus improve the membrane’s gas selectivity. Furthermore, the resulting network structure endowed the thermally treated PI membrane with excellent anti-plasticization ability. Physical characterization results show that after heat treatment, both the d-spacing and BET surface area of the PI membrane decreased, but the solvent resistance of the thermally treated PIs was significantly improved. Gas separation experiments revealed that the representative membrane (PI-PGBO-300) exhibited the optimal CO_2_/CH_4_ separation performance, with a CO_2_ permeability of 371.05 Barrer, a CO_2_/CH_4_ selectivity of 28.11, and a CO_2_ plasticization pressure exceeding 30 bar. This study provides valuable insights into the design of cross-linked polyimides (PIs) via transesterification reactions, which are capable of enhancing the performance of membrane-based gas separation processes.

## 1. Introduction

With the increasing global demand for energy structure transformation and environmental protection, gas separation membranes, characterized by low energy consumption, simple operation, and environmental friendliness, have shown great application potential in multiple gas treatment fields (such as natural gas purification, carbon capture and hydrogen recovery) [1,2]. Membrane materials are the core of membrane separation technology. Among them, polyimide (PI), as a high-performance polymer, has emerged as one of the ideal materials for gas separation membranes due to its excellent thermal stability, chemical resistance, and separation performance [3]. However, gas separation membranes face a series of urgent problems to be solved in practical applications. Firstly, they generally suffer from a “trade-off” effect between selectivity and permeability, making it difficult to meet the simultaneous demands of industrial production for high-efficiency separation and high flux [4,5]. Consequently, this limitation has become a key bottleneck restricting their performance improvement [5,6]. Secondly, conventional linear polymer membranes are prone to a plasticization issue [7]. Under high-pressure environments containing soluble gases, the selectivity and permeability of these membranes undergo a drastic reduction, which significantly impairs their separation efficiency [8].

To enhance the anti-plasticization and gas separation performance of polyimide (PI) membranes, researchers have developed various modification methods, which are mainly categorized into the following: introduction of rigid moieties (e.g., naphthalene rings, benzimidazoles, triptycene groups, spirobisindanes, ethanoanthracenes), thermal rearrangement (TR) [9], blending, and cross-linking [10]. For example, Qian Wang et al. introduced triptycene units into PI molecular chains, which increased the free volume of PI and simultaneously improved gas permeability and selectivity [11]. Shi et al. incorporated micron-sized ZIF-11 particles into a PI matrix. Compared to the pure BIMPI membrane, the MMM containing 50 wt% MZIF-11 exhibited a significant performance enhancement: the CO_2_ permeability increased by 410% to 99.0 Barrer, while the CO_2_/CH_4_ selectivity reached 60.7, representing a 35% improvement [12]. Notably, the CO_2_ plasticization pressure of this membrane exceeded 40 bar, demonstrating excellent anti-plasticization performance [13].

In addition, cross-linking is also recognized as an effective strategy for enhancing the stability and gas separation performance of PI membranes. Generally, cross-linking modification forms chemical bonds between PI molecular chains through cross-linking reactions, thus restricting the movement of chain segments and improving the stability of the entire aggregate framework. This modification method can usually improve the gas selectivity of PI membranes and simultaneously enhance the anti-plasticization performance. Up to now, common cross-linking methods include debromination cross-linking [14], decarboxylation cross-linking, and ester cross-linking. For example, Li et al. synthesized two carboxyl-containing diamines, termed CADA1 and CADA2, which were polymerized with 6FDA, BTDA, and DSDA, respectively, to form four types of PIs [15]. After thermally induced decarboxylation cross-linking, the CO_2_ permeability of the cross-linked PIs was highly improved. When the CO_2_ pressure reached up to 30 atm, no CO_2_-induced plasticization was observed in the cross-linked PIs [16]. The ester cross-linking of PI specifically refers to forming ester (–COO–) covalent bonds between PI molecular chains or between molecular chains and cross-linking agents through esterification or transesterification reactions [17]. This cross-linking process typically uses active groups such as carboxyl groups (–COOH) and hydroxyl groups (–OH) contained in PI molecular chains as reaction sites [18]. In our view, ester cross-linking plays a crucial role in regulating the microstructure (mainly the size and distribution of free volume) within the PI membrane. Consequently, this strategy enables the optimization of gas molecular transport channels and enhances the gas selectivity of the membrane. Meanwhile, it can also enhance the membrane’s anti-plasticization ability due to the formed network structure. Staudt-Bickel et al. chemically cross-linked carboxyl-containing 6FDA-mPD/DABA (9:1) PI with ethylene glycol at 250 °C for 14 h. Compared with the uncross-linked original membrane, the plasticization pressure of the cross-linked membrane increased from 14 atm to 35 atm, and the CO_2_/CH_4_ selectivity was also significantly improved [19]. Furthermore, Wind et al. studied the effects of chemical cross-linking and heat treatment on the gas permeability of PI membranes containing carboxyl groups. The results showed that chemical cross-linking with 1,4-butanediol and 1,4-cyclohexanedimethanol could increase the membrane’s plasticization pressure to 40 atm [20]. Liu et al. put forward an ester-crosslinked approach for carboxylated PIM, with PIM-1 employed as the base material. The resulting ester-crosslinked PIM-1 membrane exhibited outstanding gas separation performance that significantly surpassed the 2008 Robeson Upper Bound. Moreover, no apparent plasticization occurred even when the CO_2_ feed pressure reached as high as 4.2 MPa [21].

Numerous researchers have demonstrated that ester cross-linking can be achieved under mild conditions, typically below the glass transition temperature of PI, thereby preserving the membrane’s microporous structure and facilitating large-scale industrial production [22]. Moreover, the obtained cross-linked structure can simultaneously enhance PI membranes’ anti-plasticization performance and maintain the balance between gas permeability and selectivity [20]. Therefore, it is essential to further explore the structure-function relationship between ester cross-linking and the separation performance of PI membranes [18].

Based on these considerations, we utilized DAMBA (3,5-diamino-4-methylbenzoic acid) monomer as the precursor and synthesized two new monomers, namely DAMBO (methyl 3,5-diamino-4-methylbenzoate) and DAPGBO (3-hydroxypropyl 3,5-diamino-4-methylbenzoate), via esterification reactions. Then, we copolymerized each of these two new monomers with 4,4′-diaminodiphenylmethane (DAM) and 4,4′-(Hexafluoroisopropylidene)diphthalic anhydride (6FDA) separately to obtain the corresponding monoesterified PIs. Importantly, DAM monomer is widely used in the fabrication of high-permeability PI membranes [23]. Following this, we prepared network-structured PI materials by inducing a transesterification cross-linking reaction in the DAPGBO-based monoester PI material through thermal treatment. As expected, we found that the formation of cross-linked structures can effectively regulate the PI’s microporous structure, enhance its sieving performance, and thus improve PI membranes’ gas selectivity. Furthermore, the resulting network structure endowed the PI membrane with excellent anti-plasticization properties [24]. It is noteworthy that the thermal treatment temperature exerts a critical impact on the performance of ester-crosslinked PI membranes. Consequently, our research systematically investigated the effects of thermal treatment temperature on the molecular structure and gas separation performance of PI membranes. Additionally, we conducted a comprehensive analysis of PI materials both before and after cross-linking to evaluate their key properties, including physicochemical characteristics, morphology, thermal stability, gas separation performance, and anti-plasticization behavior.

## 2. Experimental Section

### 2.1. Chemical Materials

In this study, m-cresol (purity: 99%) was purchased from Energy Chemical (Annaiji Chemical, Shanghai, China). 4,4′-(Hexafluoroisopropylidene)diphthalic anhydride (6FDA) was obtained from Energy Chemical and dried under vacuum at 150 °C prior to use. 2,4,6-Trimethylbenzene-1,3-diamine (DAM) and 3,5-diamino-4-methylbenzoic acid (DAMBA) were both purchased from Energy Chemical and dried under vacuum at 60 °C prior to use. Anhydrous N,N-dimethylformamide (DMF), isoquinoline, and 1,3-propanediol were all procured from Energy Chemical. Anhydrous sodium carbonate, methanol, and ethyl acetate were all purchased from Tianjin Maiding Technology Co., Ltd., Tianjin, China, while concentrated sulfuric acid was acquired from Tianjin Kairuis Fine Chemical Co., Ltd., Tianjin, China. All the aforementioned reagents were used as received.

### 2.2. Synthesis of DAMBO and DAPGBO

3,5-Diamino-4-methylbenzoic acid (DAMBA) (1.0 g, 6.02 mmol) was added to a two-necked flask, and 8 mL of methanol was added. The mixture was stirred thoroughly until the solid was completely dissolved, then 1 mL of concentrated sulfuric acid was added as a catalyst. The reaction was carried out at 66 °C for 18 h. Subsequently, the mixture was allowed to cool down to room temperature, after which a saturated sodium carbonate solution was added to bring the pH within the range of 8–10. The mixture was placed in a low-temperature environment (0–4 °C) for cooling and crystallization. The target product, methyl 3,5-diamino-4-methylbenzoate (denoted as DAMBO hereinafter), was obtained by suction filtration. The product was dried in a vacuum drying oven at 70 °C for 12 h to remove moisture, and its structure was confirmed to be the target compound by proton nuclear magnetic resonance (^1^H NMR) spectroscopy. Using a similar method, 3,5-diamino-4-methylbenzoic acid 1,3-propanediol monoester (denoted as DAPGBO hereinafter) was synthesized.

### 2.3. Synthesis of PIs, Membrane Preparation, and Ester Cross-Linking via Heat Treatment

**Figure 1:**
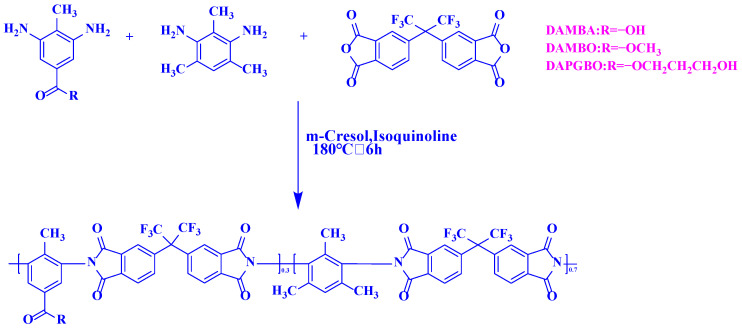
Synthesis methods of PI-MBA, PI-MBO, and PI-PGBO polymer powders.

In this work, linear PIs were synthesized via polycondensation of 6FDA, commercial diamine (DAM), and one of the four selected diamines (DABA, DAMBA, DAMBO, and DAPGBO). The resultant PIs were named PI-DABA, PI-MBA, PI-MBO, and PI-PGBO according to the selected diamines. Each selected diamine was copolymerized with DAM at a 3:7 molar ratio, and the total diamines were reacted with dianhydride (6FDA) at a 1:1 molar ratio. The PI film preparation was described in detail using PI-PGBO as a representative example. As shown in Figure 1, DAPGBO (235.2 mg, 1.05 mmol), DAM (368.1 mg, 2.45 mmol), and 6FDA (1554.9 mg, 3.5 mmol) were weighed into a two-necked flask. 8 mL of m-cresol was added, and the mixture was stirred at room temperature until almost dissolved. After adding 0.2 mL of isoquinoline, the temperature was raised to 180 °C for 6 h. After cooling, the viscous solution obtained from the reaction was poured into methanol for precipitation. This precipitation step was repeated 2–3 times to remove as much m-cresol solvent as possible. Subsequently, a Soxhlet extraction device was set up for further solvent removal. The polymer powder was obtained by suction filtration and dried in a vacuum oven at 80 °C for 24 h, yielding the final PI-PGBO product.

Films of all synthesized copolymers were prepared by the solution casting method. Specifically, a 5 wt% solid content was used: 300 mg of polymer powder was weighed and dissolved in 5 mL of DMF with sufficient stirring. The resulting solution was passed through a PTFE filter prior to being transferred into a glass vessel, and then dried in a vacuum oven at 60 °C for 48 h. Flexible and transparent PI films with a thickness of approximately 50–60 μm were obtained. The films were then peeled off from the glass substrates and dried in a vacuum oven at 100 °C for 2 h. Other PI films were prepared using a similar method. All the as-prepared films were subjected to gas permeation testing and related characterization measurements.

**Figure 2:**
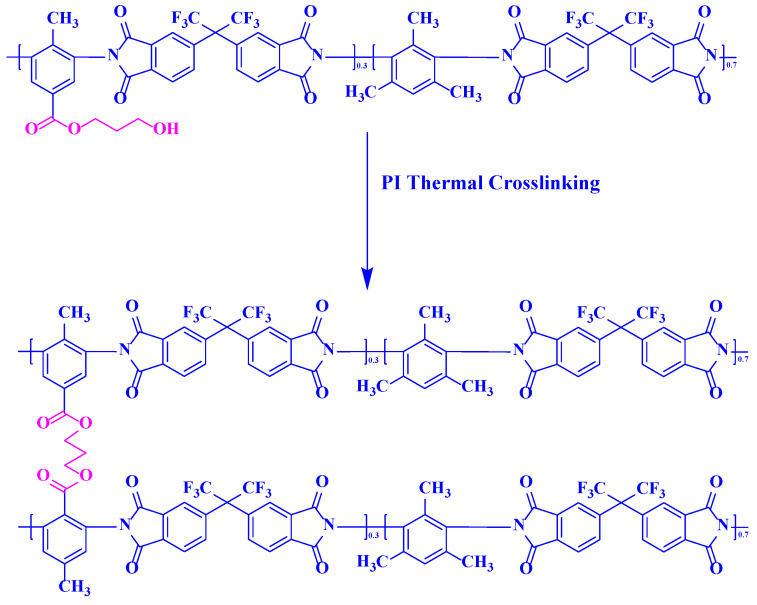
PI-PGBO polymer chains undergoing ester cross-linking reaction induced by thermal treatment.

The PI-PGBO films were heat-treated in a tube furnace (SK-G06123K) at a heating rate of 5 °C min^−1^. The specific process was as follows: the film was placed between two layers of carbon cloth; evacuation and nitrogen purging were repeated three times; then, ester cross-linking was induced at 250 °C, 300 °C, and 350 °C, respectively, for 4 h under a nitrogen atmosphere. The polymer chains obtained via the ester cross-linking reaction are shown in Figure 2. The network-structured PI films after ester cross-linking via heat treatment were named according to their heating temperatures. For example, PI-PGBO-250 refers to the PI-PGBO film heat-treated at 250 °C for 4 h.

### 2.4. Characterization and Testing

Wide-angle X-ray diffraction (WAXD) data were collected using a Rigaku Ultima IV X-ray diffractometer. The average interlayer spacing (d) was calculated according to Bragg’s Law: d = λ/(2sinθ), where λ = 1.5406 Å and θ is the X-ray diffraction angle. Fourier transform infrared (FT-IR) spectra of the PI films were acquired utilizing a PerkinElmer 782 Fourier transform spectrophotometer. The surface and cross-sectional morphologies of the films were observed using a high-resolution field-emission scanning electron microscope (SEM, Zeiss, Jena, Germany). Proton nuclear magnetic resonance (^1^H NMR) spectra were acquired on a Bruker Avance 400 spectrometer, with DMSO-d6 as the solvent. Thermogravimetric analysis (TGA) was performed using a TA Instruments Q500. All samples were first pre-dried under vacuum at 120 °C for 24 h to remove most moisture and solvents. The TGA experiments were conducted under a nitrogen flow of 50 mL/min, with the temperature ramped from 40 °C to 850 °C at a heating rate of 10 °C/min. Mechanical properties were measured using a UTM6103 electronic universal testing machine (Shenzhen SANS Material Testing Co., Ltd., Shenzhen, China). Test samples were cut into strips with a width of 3 mm and a length of 10 mm, then stretched to break at a displacement rate of 3 mm/min. The tensile strength and elongation at break were recorded. Adsorption experiments were carried out at 77 K using a Micromeritics ASAP 2460 2.02 surface area and porosity analyzer. Nitrogen adsorption behavior of the samples was measured to obtain the corresponding adsorption–desorption isotherms and specific surface area. The gel fraction was determined by measuring the mass of the film before and after immersion in DMF solvent, using the formula: Gel fraction = (Mass of film after immersion/Mass of film before immersion) × 100%.

## 3. Results and Discussion

### 3.1. Synthesis and Characterization of Monomers DAMBO, DAPGBO, and Corresponding PI

**Figure 3:**
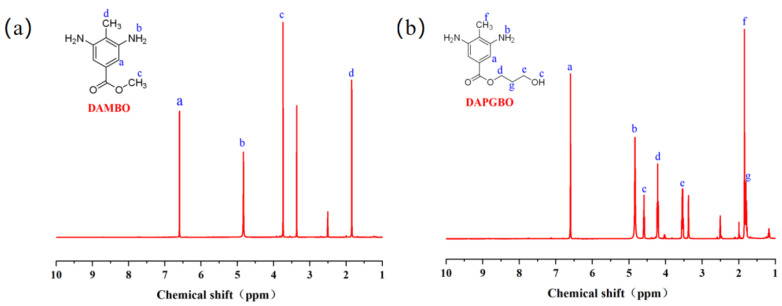
^1^H NMR spectrum of DAMBO (**a**) and ^1^H NMR spectrum of DAPGBO (**b**).

In this study, PI powders were synthesized via the copolymerization of 6FDA, DAM, and one of the selected diamine monomers (DABA, DAMBA, DAMBO, DAPGBO). The proton nuclear magnetic resonance (^1^H NMR) spectra of DAMBO and DAPGBO monomers are shown in Figure 3a and Figure 3b, respectively. The analysis results of the positions of each characteristic peak are consistent with the monomer structural formulas. Specifically, in the DAMBO structure, the methyl protons of the methyl ester group (–COOCH_3_) exhibit a characteristic peak around 3.7–3.9 ppm, confirming the successful conversion of carboxyl groups to methyl esters. Regarding DAPGBO monomer, the peaks ranging from 2.7 to 4.5 ppm can be assigned to the alkyl protons in the side chain. In addition, the appearance of a new peak located at 4.6 ppm could be assigned to the proton of the hydroxyl group. All these results confirmed that the esterification reaction between DAMBA and 1,3-propanediol occurred.

**Figure 4:**
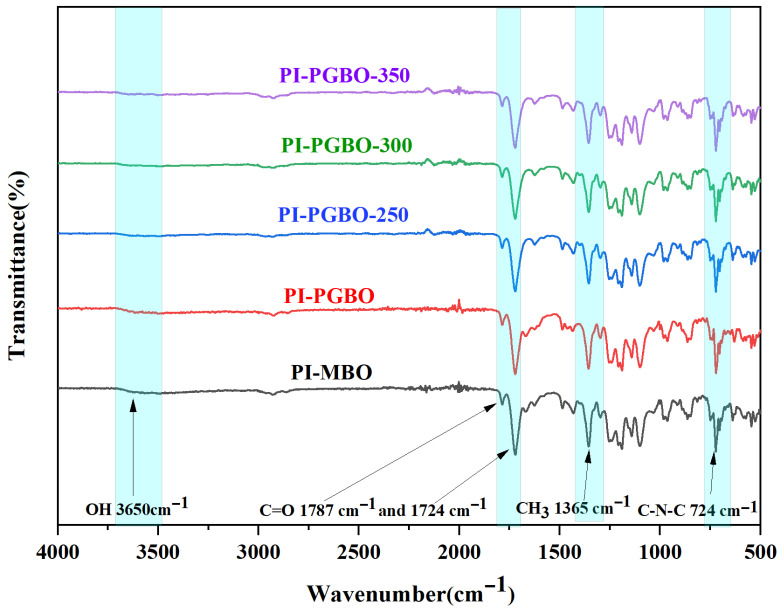
FT-IR spectra of PI-MBO, PI-PGBO, and PI-PGBO membranes treated at different temperatures.

FT-IR analysis was conducted on the membranes fabricated from PI-MBO and PI-PGBO at different heating temperatures. The results are shown in Figure 4 below. As expected, all the prepared membranes exhibit distinct absorption bands corresponding to imide groups at specific wavenumbers: 724 cm^−1^ (imide bending), 1724 cm^−1^ (symmetric stretching of C=O), and 1787 cm^−1^ (asymmetric stretching of C=O) [25]. Furthermore, this typical PI structure remained unchanged after heat treatment at 250 °C, 300 °C, and 350 °C, proving that the main backbone of PI has not changed significantly [26]. In addition, all the membrane samples display characteristic peaks of –CH_3_ at 1365 cm^−1^.

To investigate the effect of cross-linking on the chemical structure of the membranes, FT-IR analysis was performed on samples subjected to heat treatment at different temperatures for 4 h. As shown in the figure, the absorption peak of alcoholic hydroxyl groups in PI-PGBO appears in the range of 3500–3590 cm^−1^ [27]. Upon closer inspection, it was found that as the cross-linking temperature rose, the intensity of the hydroxyl peak within the range of 3500–3590 cm^−1^ for the thermally treated PI-PGBO samples tended to decrease. This confirms that PI-PGBO underwent esterification cross-linking reactions at different heating temperatures, leading to a significant decrease in the intensity of the –OH absorption peak.

To further prove the occurrence of the cross-linking reaction, both the uncross-linked PI-PGBO membranes and the thermally treated PI-PGBO membranes were immersed in N,N-dimethylformamide (DMF) to investigate their degree of cross-linking [28]. The gel fraction data are presented in Table 1 below. According to the gel fraction test, both PI-MBO and PI-PGBO samples were completely soluble in DMF with a gel fraction of 0%, indicating the absence of cross-linking in their structures. After cross-linking at 250 °C, 300 °C, and 350 °C for 4 h, the samples were no longer completely soluble in DMF, and the degree of cross-linking gradually increased with rising temperature. PI-PGBO-300 was almost insoluble in DMF, exhibiting the highest degree of cross-linking with a calculated gel fraction of 100%, which indicates the formation of a highly cross-linked structure. However, when the temperature increased to 350 °C, the gel fraction decreased instead. A possible reason is that partial ester groups within the PI-PGBO treated at 350 °C decomposed [29]. Therefore, this undermined the stability of the network structure of PI-PGBO-350, resulting in a decrease in the gel fraction.

Furthermore, a scanning electron microscope (SEM) was used to observe the surfaces and cross-sections of the prepared samples, including PI-MBO membranes and PI-PGBO membranes, with the results presented in Figure S1. The results indicate that all the prepared films exhibit a smooth and dense morphology without obvious defects [30].

### 3.2. X-Ray Diffraction (XRD) Analysis of Membranes

**Figure 5:**
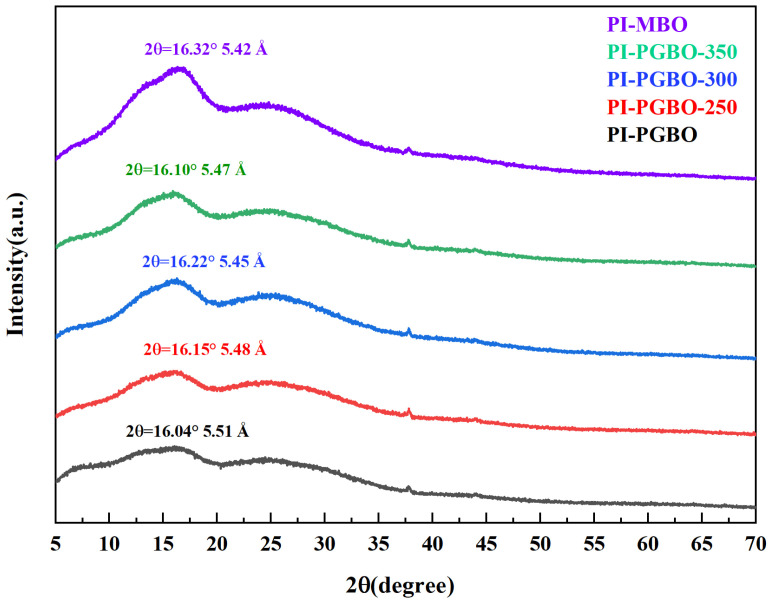
WAXD curves of PI-MBO, PI-PGBO, and PI-PGBO membranes treated at different temperatures.

Figure 5 displays the wide-angle X-ray diffraction (WAXD) patterns of all prepared membrane materials. It can be observed that all membranes exhibit two broad diffraction peaks centered around 2θ = 16° and 2θ = 25°, indicating their amorphous state. Typically, the average interchain distance (referred to as d-spacing) in polymers can be calculated using Bragg’s law (2dsinθ = nλ). Focusing on the main diffraction peak (near 2θ = 16°): compared with PI-MBO (2θ = 16.32°, d = 5.42 Å), the main diffraction peak of PI-PGBO shifts to a lower angle at 2θ = 16.04°, with its d-spacing increasing to 5.51 Å. This phenomenon can be attributed to the bulky occupying effect of the long side chains in PI-PGBO: the voluminous side groups disrupt the tight packing of the polymer chains, thereby reducing the diffraction angle, shifting the peak to a lower angle, and increasing the interchain distance.

After thermal treatment at different temperatures, the main diffraction peak of PI-PGBO progressively shifted to higher angles: for PI-PGBO-250, the main diffraction peak was located at 2θ = 16.15° (d = 5.48 Å); for PI-PGBO-300, it shifted to 2θ = 16.22° (d = 5.45 Å); and for PI-PGBO-350, it was at 2θ = 16.10° (d = 5.47 Å). Within the 250–300 °C range, the diffraction peak shifted toward larger 2θ values with increasing temperature, indicating the formation of a compact network structure due to ester cross-linking, which resulted in tighter molecular chain packing and a consequent reduction in d-spacing [31]. Among the cross-linked membranes, PI-PGBO-300 exhibited the largest diffraction angle (2θ = 16.22°) and the corresponding smallest d-spacing (5.45 Å), suggesting the highest degree of cross-linking, consistent with the gel content measurements and confirming that the ester-exchange cross-linking reaction was essentially complete at 300 °C. When the temperature was raised to 350 °C, the main diffraction peak shifted slightly to a lower angle (2θ decreased to 16.10°) and the d-spacing slightly increased to 5.47 Å, which can be attributed to partial decomposition of the PI side chains at elevated temperatures, leading to a slight loosening of the molecular chain packing [32].

### 3.3. Thermogravimetric (TGA) and Derivative Thermogravimetric (DTG) Tests of Membranes

**Figure 6:**
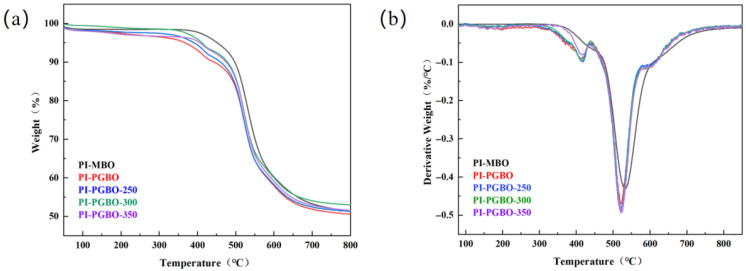
TGA curves (**a**) and DTG curves (**b**) of PI-MBO, PI-PGBO, and PI-PGBO membranes treated at different temperatures.

The thermal stability of PI-MBO, PI-PGBO membranes, and the thermally treated PI-PGBO membranes was investigated via thermogravimetric analysis (TGA) and derivative thermogravimetry (DTG), with the results shown in Figure 6a and Figure 6b, respectively. All the prepared membranes exhibit the first weight loss in the temperature range of 375 –425 °C, which may be caused by the cleavage of ester groups in the side chains [33]. A second weight loss occurred at 550 °C for all membranes, resulting from the cleavage of the polyimide (PI) polymer main chain.

Concerning the PI-PGBO and its thermally treated membranes (PI-PGBO-250, PI-PGBO-300, and PI-PGBO-350), as the cross-linking temperature gradually increased, the first weight loss phenomenon in the range of 375–425 °C became increasingly less significant. This is because the thermal treatment initiates the ester cross-linking reaction, which can significantly enhance the thermal stability of polyimide membranes [34,35]. It is worth noting that the PI-PGBO-300 membrane exhibits the best thermal stability, with a 5% weight loss temperature (T_d,5%_) of 410 °C and a residual carbon rate of 53.02%, showing the optimal thermal stability [36]. In comparison, conventional aromatic polyimides typically exhibit T_d,5%_ values in the range of 500–600 °C, whereas functionalized or side-chain-modified PIs often show a lower degradation onset temperature due to the introduction of thermally labile groups [37]. The observed T_d,5%_ of ~410 °C for PI-PGBO-300, is comparable to that of other ester-cross-linked polyimide systems reported in the literature. For instance, cross-linked polyimide membranes derived from thermal treatment at 300 °C displayed T_d,5%_ around 400–420 °C [38].

In the case of the PI-PGBO-350 membrane, although it underwent high-temperature heat treatment, its thermal stability was lower than that of PI-PGBO-300. This phenomenon can be attributed to the disruption of the network structure caused by excessively high temperatures, which subsequently impairs thermal stability [39]. Similar observations have been reported for thermally cross-linked polyimides, in which treatment above an optimal temperature leads to partial degradation of cross-links or side-chain decomposition, resulting in decreased thermal stability. These findings are consistent with the preliminary results from gel fraction measurements and X-ray diffraction (XRD) analysis reported earlier. The overall thermal behavior of the PI-PGBO series aligns with the general trend that moderate cross-linking enhances thermal stability, whereas excessive treatment can induce structural deterioration [40]. These results highlight the importance of optimizing the cross-linking temperature to achieve a balance between improved thermal stability and maintained structural integrity.

### 3.4. BET Test and Mechanical Property Test

**Figure 7:**
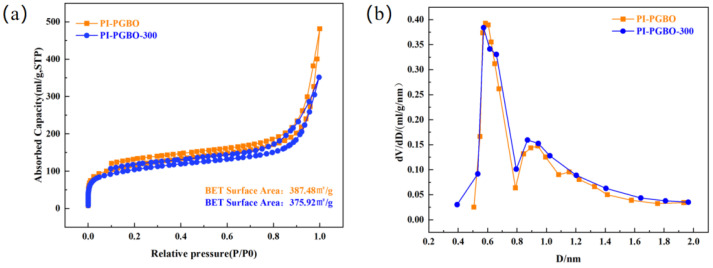
N_2_ adsorption–desorption isotherms (**a**) and H-K method-derived pore size distributions (**b**) of PI-PGBO and PI-PGBO-300.

To investigate the effect of ester cross-linking on the microporous structure of PI-PGBO and PI-PGBO-300, nitrogen adsorption–desorption isotherms of the PI membranes were tested at 77 K, and then the microporous characteristics of both membranes were analyzed. As shown in Figure 7a, compared with the original PI-PGBO membrane, the BET specific surface area of PI-PGBO-300 slightly decreased from 387.48 m^2^/g to 375.92 m^2^/g. This is mainly due to the ester cross-linking reaction, which reduces the molecular chain spacing of the membrane material and further decreases the micropore volume inside the membrane. This result is consistent with the variation law of d-spacing obtained from X-ray diffraction (XRD) analysis.

Moreover, the Horvath-Kawazoe (H-K) method was used to determine the corresponding pore size distribution of PI-PGBO and PI-PGBO-300. As shown in Figure 7b. Generally, pores with a pore size less than 0.7 nm are defined as ultra-micropores [41]. A substantial body of research has established that the concentration of ultra-micropores is highly significant for boosting the membrane’s gas selectivity. Although the pore size distributions of PI-PGBO and PI-PGBO-300 are very similar, PI-PGBO-300 exhibits smaller ultra-micropores in the range of 0.38–0.5 nm. This indicates that after thermal treatment at 300 °C, an increased content of ultra-micropores was formed in PI-PGBO-300 [42]. Therefore, it can be inferred that the thermally treated PI-PGBO-300 membrane may have excellent gas selectivity.

**Figure 8:**
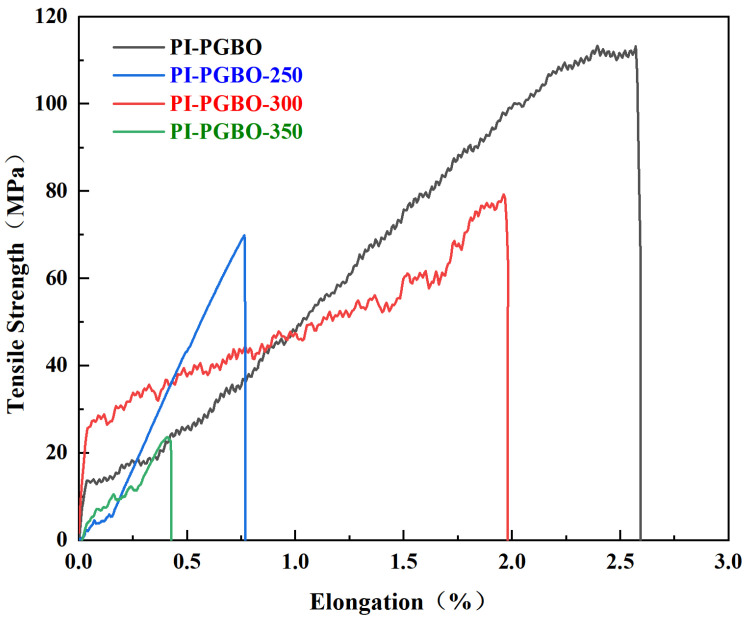
Tensile curves of PI-PGBO films and PI-PGBO films treated at different temperatures.

Figure 8 presents the tensile stress–strain curves of as-prepared PI-PGBO films and those subjected to heat treatment at different temperatures, where all samples exhibit the typical fracture behavior of brittle polymers [43]. Mechanical property data reveal that the as-prepared PI-PGBO film achieves the optimal mechanical performance with a tensile strength of 113 MPa and an elongation at break of 2.55%. For PI-PGBO-250, a sharp increase in brittleness was observed, with the elongation at break decreasing to 0.8% and the tensile strength dropping below 70 MPa. In contrast, PI-PGBO-300 treated at 300 °C exhibited recovered mechanical properties, featuring a tensile strength of 78 MPa and an elongation at break of 1.91%. Whereas PI-PGBO-350, subjected to 350 °C heat treatment, displayed the poorest mechanical performance, fracturing at a strain of less than 0.5% [44]. These results indicate that heat treatment temperature precisely regulates the mechanical behavior of PI-PGBO films by modulating the molecular chain structure [45]. Heat treatment at 300 °C can facilitate the complete imidization reaction and simultaneously induce moderate orientation and crosslinking of molecular chains, thereby exhibiting superior comprehensive mechanical properties, whereas heat treatment at 350 °C may cause excessive crosslinking or even degradation of molecular chains, completely destroying the mechanical integrity of the film and ultimately resulting in brittle fracture at low strain. This phenomenon is consistent with existing research conclusions that ester crosslinking enhances the brittleness of polymer materials [46], and also provides a mechanical property basis for the application of PI-PGBO-300 in fields such as flexible electronic substrates and high-temperature resistant films [47].

### 3.5. Pure Gas Separation Performance

In this study, we systematically tested the prepared polyimide membranes (PI-MBA, PI-MBO, and PI-PGBO) using the time-lag method. The tests were carried out under the conditions of 35 °C and 2 bar. We investigated their permeation performance with respect to four pure gases (CH_4_, N_2_, O_2_, and CO_2_) and their selective separation performance for three gas pairs (O_2_/N_2_, CO_2_/N_2_, and CO_2_/CH_4_). Among them, PI-MBA was used for the comparative research. The gas separation performance of various membrane materials is shown in Table 2 below. The experimental results showed that, based on the inverse relationship with the kinetic diameter of gas molecules (molecular diameter: CH_4_ > N_2_ > O_2_ > CO_2_), the gas permeability of all tested membranes exhibited a consistent order: P(CH_4_) < P(N_2_) < P(O_2_) < P(CO_2_) [48].

When compared to the PI-MBA membrane, the PI-MBO membrane that underwent methanol esterification modification exhibited a notable rise in CO_2_ permeability. It reached 360.59 Barrer, which represented a 68% increase. Meanwhile, the CO_2_/CH_4_ selectivity decreased by only 13%, reaching 26.13. To further enhance the comprehensive gas separation performance of the membrane, 1,3-propanediol was employed for monoesterification modification to prepare the PI-PGBO membrane. Compared with the PI-MBA, the CO_2_ permeability of the PI-PGBO membrane modified by 1,3-propanediol esterification increased by 118%, reaching 476.56 Barrer. As we know, the introduction of bulky pendant groups into the PI backbone reduces the segmental packing density [49]. This leads to the formation of more micropores inside the membrane and makes the molecular chain packing looser. As a result, the gas permeability of PI-MBO and PI-PGBO is significantly increased relative to that of PI-MBA. Meanwhile, limited by the trade-off effect, we also found that while achieving high gas permeability, the gas selectivities of PI-MBO and PI-PGBO would decline to some extent.

Next, the PI-PGBO was chosen for further thermal treatment due to its capacity for an ester cross-linking reaction. When the temperature increased from 250 °C to 300 °C, we could find that although the gas permeability of thermally treated PI-PGBO slightly decreased, its gas selectivity was increased obviously due to the rising cross-linking density of the PI-PGBO membrane with the rise in temperature [50]. For example, after the heat treatment at 300 °C, the CO_2_/CH_4_ selectivity of the PI-PGBO-300 membrane increased from 23.05 to 28.11, which was a 22% increase compared with the PI-MBA membrane. However, when the temperature exceeded 300 °C, the PI structure began to degrade, leading to an increase in CO_2_ permeability and a decrease in gas selectivity. For instance, the CO_2_/CH_4_ selectivity for PI-PGBO-350 decreased from 28.11 to 23.50 [51]. Overall, despite the slight reduction in gas permeability induced by 1,3-propanediol-based ester cross-linking modification, this strategy remarkably improves membrane selectivity, a property that is pivotal to the practical utilization of PI membranes.

**Figure 9:**
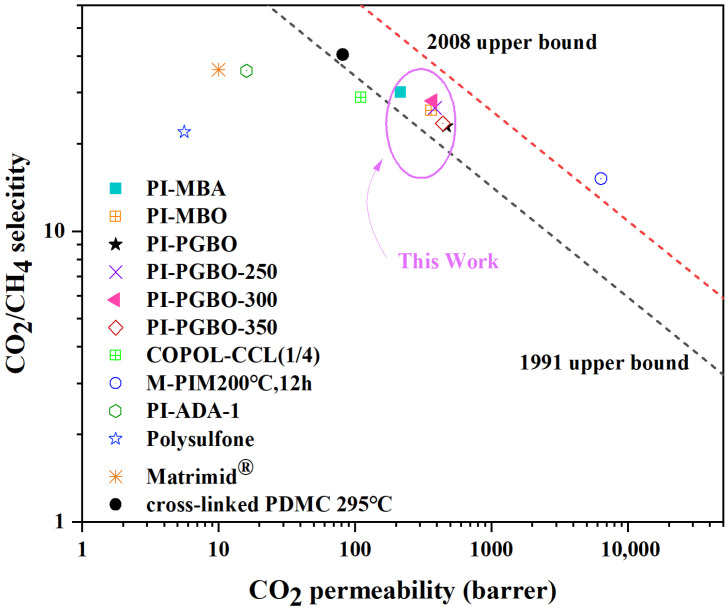
Relationships between permeability and selectivity of prepared membranes, and the 1991 and 2008 Robeson Upper Bounds for the CO_2_/CH_4_ system.

The “Upper Bound” is currently a widely recognized standard for evaluating the permeability and selectivity of membrane materials [52], as it quantitatively defines the trade-off relationship between gas permeability and selectivity of membrane materials. Figure 9 illustrates the relationship between CO_2_ permeability and CO_2_/CH_4_ selectivity for the reported base membranes and the polyimide (PI) membranes prepared in this study. For the PI-PGBO-300 membrane developed herein, its CO_2_ permeability reaches 371.05 Barrer, with a CO_2_/CH_4_ selectivity of 28.11. Its performance falls between the 1991 and 2008 Robeson Upper Bound curves. This directly confirms that its comprehensive separation performance lies between the 1991 and 2008 Robeson Upper Bounds [53], and it significantly exceeds the selectivity level defined by the Upper Bounds at the corresponding permeability.

In addition, compared with commercial separation membranes, polysulfone has a CO_2_ permeability of 5.6 Barrer and a CO_2_/CH_4_ selectivity of 22, while Matrimid^®^ has a permeability of 10 Barrer and a selectivity of 36 [54,55]. Although Matrimid^®^ has slightly higher selectivity, the CO_2_ permeability of PI-PGBO-300 is 37.1 times that of Matrimid^®^, demonstrating a more balanced performance of high permeability and high selectivity. Compared with other cross-linked membranes, the PDMC membrane cross-linked at 295 °C has a CO_2_ permeability of 81.2 Barrer and a CO_2_/CH_4_ selectivity of 40.6 [50]. The CO_2_ permeability of PI-PGBO-300 is approximately 4.6 times that of the PDMC membrane, while the selectivity of PI-PGBO-300 reaches 28.11, achieving a more excellent balance between permeability and selectivity. This quantitative analysis fully demonstrates that the PI-PGBO-300 membrane has distinct advantages in comprehensive gas separation performance, and its permeability-selectivity trade-off relationship breaks through the limitations of traditional Upper Bounds at the corresponding permeability level.

### 3.6. Anti-Plasticization Performance and Aging Performance

**Figure 10:**
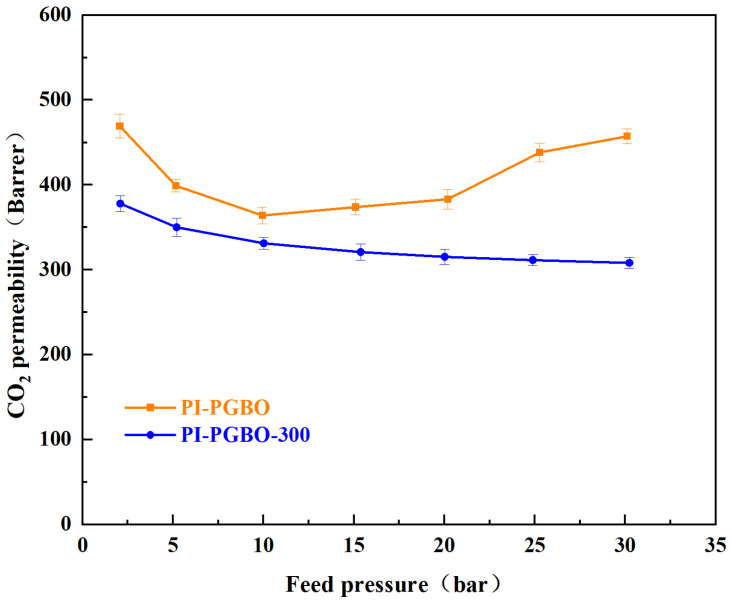
Plasticization resistance of PI-PGBO and PI-PGBO-300.

As a condensable gas, CO_2_ can unexpectedly increase the mobility of polymer chains and expand the free volume under high-pressure conditions, and such a phenomenon is referred to as the “plasticization effect” [56]. This phenomenon typically impairs the sieving performance of PI membranes, consequently resulting in a substantial reduction in their selective separation capability. Figure 10 shows the CO_2_ permeability of the PI-PGBO and PI-PGBO-300 under different feed pressures ranging from 2 to 30 bar. It can be seen that the inflection point of gas permeability for the PI-PGBO membrane appears at 10 bar, indicating that the plasticization effect has occurred around this pressure point. However, after thermally induced ester-crosslinking, the PI-PGBO-300 membrane forms a highly interconnected three-dimensional network structure [57]. Even at a CO_2_ pressure as high as 30 bar, no significant plasticization behavior is detected. The results demonstrate that the ester-crosslinked PI-PGBO-300 membrane exhibits superior anti-plasticization performance [57].

A 180-day aging test was performed on two polyimide membranes, PI-PGBO and thermally crosslinked PI-PGBO-300. As shown in Table 3, the CO_2_ permeability of PI-PGBO decreased from 467.56 to 284.68 Barrer, while its CO_2_/CH_4_ selectivity increased from 23.05 to 26.26. Similarly, PI-PGBO-300 showed a decline in CO_2_ permeability from 371.05 to 208 Barrer, accompanied by an increase in CO_2_/CH_4_ selectivity from 28.11 to 29.25. The reduction in gas permeability with prolonged aging time is likely attributed to physical aging and structural relaxation of the polymer matrix, which reduces free volume and restricts chain segment mobility. Notably, despite the decline in permeability, the gas selectivity of both membranes improved gradually with aging time, indicating that the materials retain strong molecular sieving ability after long-term aging. Furthermore, PI-PGBO-300 exhibited higher initial selectivity and more stable aging performance compared with PI-PGBO, suggesting that thermal crosslinking effectively enhances the structural stability and long-term separation consistency of the membrane. These results demonstrate that PI-PGBO-300 possesses superior aging resistance and is more suitable for long-term gas separation applications.

## 4. Conclusions

In this study, we first prepared the monoesterified PIs, namely PI-MBO and PI-PGBO, via a copolycondensation reaction. Compared with the PI-MBO membrane, the CO_2_ permeability of the PI-PGBO membrane modified by 1,3-propanediol esterification increased by 118%, reaching 476.56 Barrer due to the steric effect of large-volume side groups. Subsequently, we carried out thermal treatment on the PI-PGBO membrane to trigger a transesterification cross-linking reaction, thereby further preparing the cross-linked PIs at different heating temperatures. Gel fraction results demonstrated that after cross-linking at 250 °C, 300 °C, and 350 °C, the thermally treated PI-PGBO membranes were no longer completely soluble in DMF, and the degree of cross-linking gradually increased with rising temperature. Compared with PI-PGBO, both the d-spacing value (5.51 Å vs. 5.45 Å) and the BET specific surface area (387.48 m^2^/g vs. 375.92 m^2^/g) decreased for PI-PGBO-300 due to the transesterification cross-linking reaction. The membrane named PI-PGBO-300 demonstrated the best CO_2_/CH_4_ separation performance with a CO_2_ permeability of 371.05 Barrer and a CO_2_/CH_4_ selectivity of 28.11. Meanwhile, even when the CO_2_ pressure reached as high as 30 bar, no obvious plasticization was observed for PI-PGBO-300. However, we found that after thermal treatment and cross-linking reaction, the mechanical properties of the PI membrane decreased significantly, which is not conducive to further processing and molding of the PI membrane material. Thus, it is also the focus of our future optimization work. In general, this research provides insights into the structural design of novel cross-linked polyimide (PI) materials through the transesterification cross-linking reaction. Particularly, it focuses on PI membranes that possess excellent selectivity and plasticization resistance.

## Figures and Tables

**Table 1:** Diffraction angle (2θ), d-spacing, thermal properties, and gel fraction of PI-MBO, PI-PGBO, and PI-PGBO membranes thermally treated at different temperatures.

Polyimides	2θ (°)	d-Spacing (Å)	T_d,5%_ (°C)	Thermalstability	Gelfraction
PI-MBO	16.32	5.42	445	51.58	0%
PI-PGBO	16.04	5.51	366	50.62	0%
PI-PGBO-250	16.15	5.48	390	51.38	91.5%
PI-PGBO-300	16.22	5.45	410	53.02	100%
PI-PGBO-350	16.10	5.47	405	51.59	82.6%

**Table 2:** Pure gas permeabilities and selectivities of PI-MBO, PI-PGBO, and PI-PGBO membranes thermally treated at different temperatures.

Polyimides	Permeability (Barrer)				Selectivity (Px/y)		
	CH_4_	N_2_	O_2_	CO_2_	PO_2_/N_2_	PCO_2_/N_2_	PCO_2_/CH_4_
PI-MBA	7.87 ± 0.21	9.47 ± 0.35	44.44 ± 1.22	214.5 ± 5.80	4.69 ± 0.15	22.65 ± 0.78	30.15 ± 0.95
PI-MBO	13.80 ± 0.42	19.01 ± 0.61	76.84 ± 2.10	360.59 ± 8.95	4.04 ± 0.12	18.96 ± 0.55	26.13 ± 0.72
PI-PGBO	20.28 ± 0.65	27.36 ± 0.88	90.76 ± 2.55	467.56 ± 11.20	3.32 ± 0.10	17.09 ± 0.48	23.05 ± 0.62
PI-PGBO-250	14.40 ± 0.40	19.14 ± 0.59	74.77 ± 2.05	384.66 ± 9.50	3.91 ± 0.11	20.10 ± 0.60	26.63 ± 0.75
PI-PGBO-300	13.20 ± 0.38	17.03 ± 0.52	68.96 ± 1.85	371.05 ± 8.85	4.05 ± 0.13	21.79 ± 0.65	28.11 ± 0.80
PI-PGBO-350	18.70 ± 0.55	24.70 ± 0.75	84.76 ± 2.30	439.71 ± 10.50	3.73 ± 0.10	17.80 ± 0.52	23.50 ± 0.65
COPOL-CCL (1/4)	3.8	5.4	26	110	4.9	20.4	29
M-PIM200 °C,12 h	416	302	984	6347	3.3	21.0	15.2
PI-ADA-1	0.45	0.83	4.79	16.06	5.77	19.34	35.7
Polysulfone	0.25	0.25	1.4	5.6	5.6	22.4	22
Matrimid^®^	0.28	0.32	2.1	10	6.6	31	36
cross-linked PDMC 295 °C	2.0	-	-	81.2	-	-	40.6

Footnote: All data in this table are presented as the mean ± standard deviation (SD) of three independent replicate experiments conducted under identical conditions.

**Table 3:** Aging performance of PI-PGBO and PI-PGBO-300.

Polyimides	Permeability (Barrer)				Selectivity (Px/y)		
	CH_4_	N_2_	O_2_	CO_2_	PO_2_/N_2_	PCO_2_/N_2_	PCO_2_/CH_4_
PI-PGBO–0 days	20.28 ± 0.65	27.36 ± 0.88	90.76 ± 2.55	467.56 ± 11.20	3.32 ± 0.10	17.09 ± 0.48	23.05 ± 0.62
PI-PGBO–30 days	19.44 ± 0.58	23.22 ± 0.70	84.76 ± 2.54	456.30 ± 13.68	3.65 ± 0.11	19.65 ± 0.59	23.47 ± 0.70
PI-PGBO–90 days	17.11 ± 0.51	20.15 ± 0.60	75.63 ± 2.27	435.07 ± 13.05	3.75 ± 0.11	21.59 ± 0.65	25.43 ± 0.76
PI-PGBO–180 days	10.84 ± 0.32	12.94 ± 0.39	50.85 ± 1.53	284.68 ± 8.54	3.93 ± 0.12	22.00 ± 0.66	26.26 ± 0.79
PI-PGBO-300–0 days	13.20 ± 0.38	17.03 ± 0.52	68.96 ± 1.85	371.05 ± 8.85	4.05 ± 0.13	21.79 ± 0.65	28.11 ± 0.80
PI-PGBO-300–30 days	12.63 ± 0.38	16.28 ± 0.49	66.65 ± 2.00	359 ± 10.77	4.09 ± 0.12	22.05 ± 0.66	28.42 ± 0.85
PI-PGBO-300–90 days	9.82 ± 0.29	12.75 ± 0.38	54.32 ± 1.63	283.5 ± 8.51	4.23 ± 0.13	22.28 ± 0.67	28.87 ± 0.87
PI-PGBO-300–180 days	7.11 ± 0.21	9.22 ± 0.28	42.66 ± 1.28	208 ± 6.24	4.17 ± 0.13	22.56 ± 0.68	29.25 ± 0.88

Footnote: All data in this table are presented as the mean ± standard deviation (SD) of three independent replicate experiments conducted under identical conditions.

## Data Availability

The original contributions presented in this study are included in the article/Supplementary Materials. Further inquiries can be directed to the corresponding author.

## Supplementary Materials

The following supporting information can be downloaded at: https://www.mdpi.com/article/10.3390/membranes16010047/s1, Figure S1: SEM images of various membranes; Figure S2: Robeson Upper Bound Graph for CO_2_/N_2_ of Various Membranes; Table S1: Data on plasticization resistance of PI-PGBO and PI-PGBO-300.
