# Supplementary material for: Preparation of Ester-Crosslinked PI Membranes with Enhanced Gas Selectivity and Plasticization Resistance
Source: Membranes (Basel). 2026 Jan 20;16(1):47. doi: 10.3390/membranes16010047 (PMC12844043; doi:10.3390/membranes16010047)
Supplement: Supplementary file 1 [file membranes-16-00047-s001.zip › membranes-4007482-supplementary.pdf]

Supporting information for

Preparation of ester-crosslinked PI membranes with enhanced gas selectivity and plasticization resistance

Yu Li, Jiangzhou Luo, Honglei Ling, Song Xue**

School of Chemistry & Chemical Engineering, Tianjin Key Laboratory of Organic Solar Cells and Photochemical Conversion, Tianjin University of Technology, Tianjin, 300384, China

* Corresponding authors: luojz_tjut@163.com;xuesong@ustc.edu.cn

Content

1. **Pure Gas Testing Equations and Parameters**
2. **Scanning Electron Micrographs (SEM Images) of Various Membranes**
3. **Robeson Upper Bound Graph for CO_2/N_2 of Various Membranes**
4. **Data on plasticization resistance of PI-PGBO and PI-PGBO-300**
5. **References**

1. Pure Gas Testing Equations and Parameters

Gas permeability of the fabricated membrane towards four pure gases (CH_4, N_2, O_2, and CO_2, tested sequentially as listed) was systematically characterized via the constant volume/variable time delay method. During the experimental measurements, the membrane's permeation behavior—quantified by the downstream pressure-time profile—was continuously monitored using an Inficon sensor with a sensitivity range spanning 0–10 Torr[1]. The instrumental leak rate was determined to be approximately 2.07 × 10^{-6} Torr/sec, with the downstream volume of the test system measured as ~24.9 cm^3[2]. Gas permeability of the membrane was further derived utilizing the following mathematical expression.

$$P = D \times S = 10^{10} \times \frac{V_d \times l}{p_{up} \times T \times R \times A} \times \frac{dp}{dt} \qquad (1)$$

Herein, P denotes the gas permeability (Barrer), with the unit definition specified as 1 Barrer = 10^{-10} cm^3 (STP)·cm·cm^{-2}·s^{-1}·$cmHg^{-1}$. Vd represents the calibrated downstream volume (cm^3), l stands for the membrane thickness (cm), and pup refers to the upstream pressure (cmHg). A corresponds to the effective membrane area (cm^2), T denotes the operating temperature (K), and R is the universal gas constant with a value of 0.278 cm^3·cmHg·cm^{-3}(STP)·K^{-1}. Additionally, dp/dt denotes the steady-state pressure increment in the downstream side (cmHg·s^{-1}).

The diffusion coefficient (D, cm^2·s^{-1}) for the fabricated polymer membrane was further determined via the following approach.

$$D = l^2/6\theta \qquad (2)$$

Herein, l denotes the membrane thickness, while θ represents the permeation time lag derived from the gas permeability measurements.

The gas solubility coefficient (S, cm^3(STP)·cm^{-3}·$cmHg^{-1}$) was further derived based on the established correlation.

$$S = P/D \qquad (3)$$

The ideal gas selectivity ($P_{X/Y}$) is defined as the ratio of the individual gas permeabilities ($P_{X/Y} = P_X/P_Y$), where P_X and P_Y correspond to the gas permeabilities of gases X and Y, respectively. Triplicate measurements were performed for each

membrane type, yielding relative deviations of ~5% for both gas permeability and selectivity.

$$P_{X/Y} = P_X / \mathrm{P}_Y \tag{4}$$

2. Scanning Electron Micrographs (SEM Images) of Various Membranes

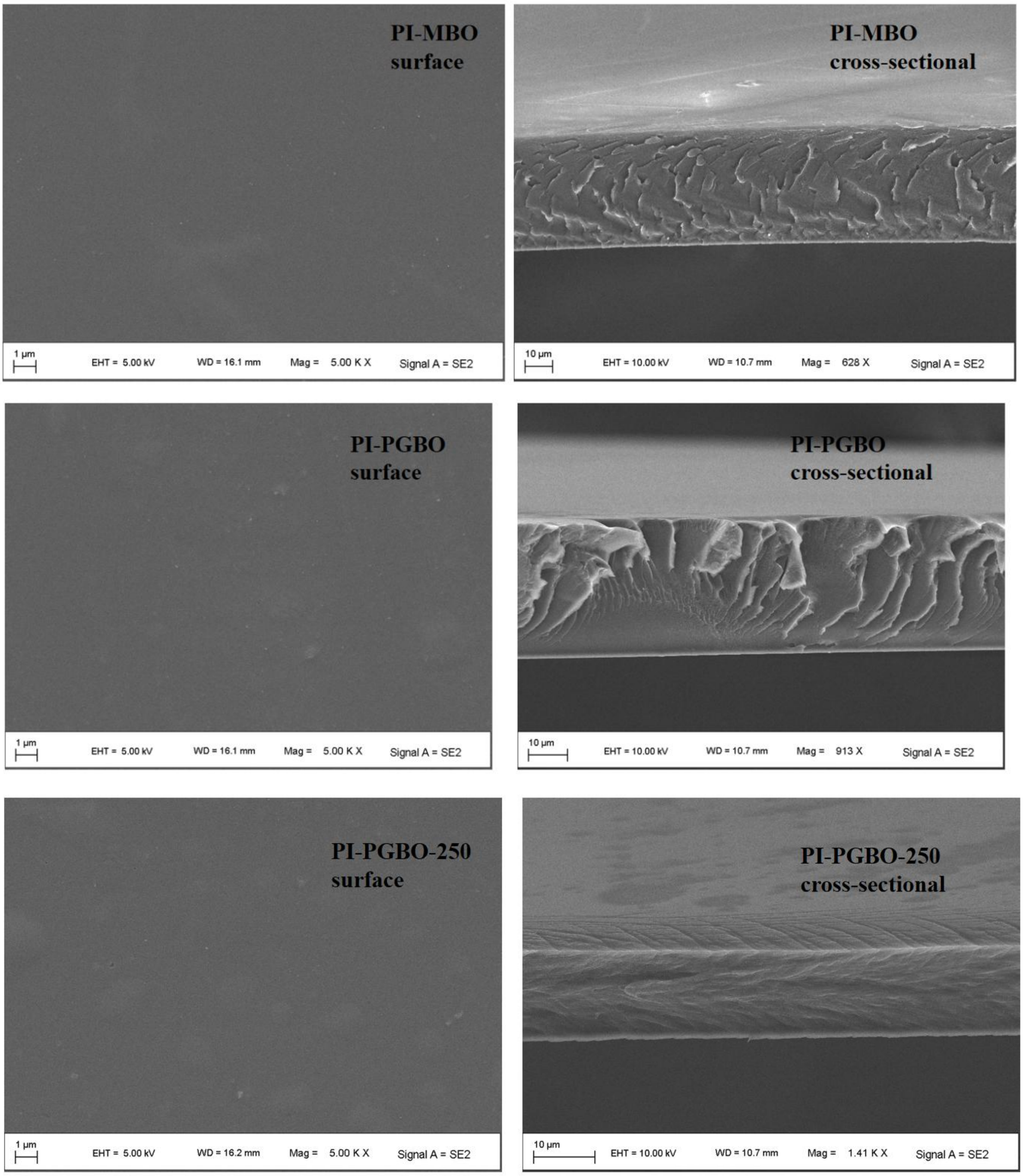

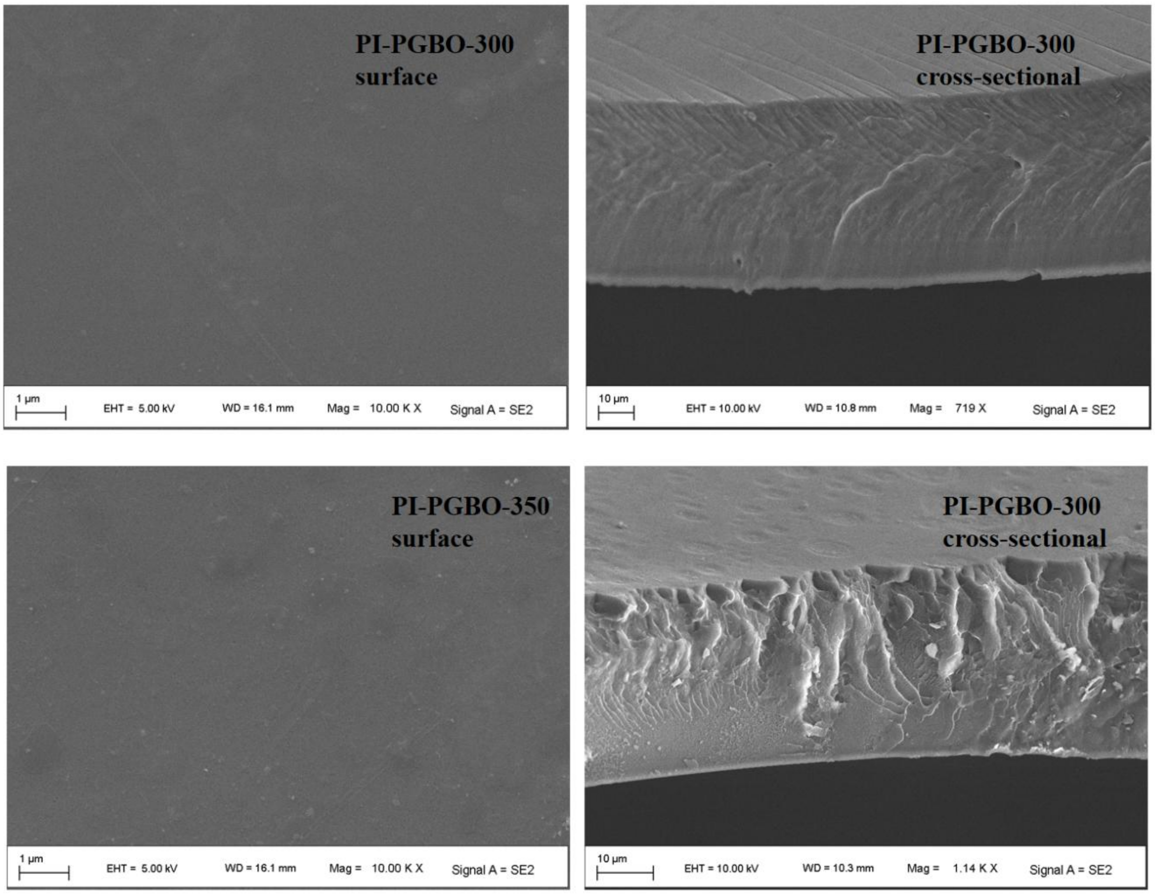

Fig. S1. SEM images of various membranes.

3. Robeson Upper Bound Graph for CO_2/N_2 of Various Membranes

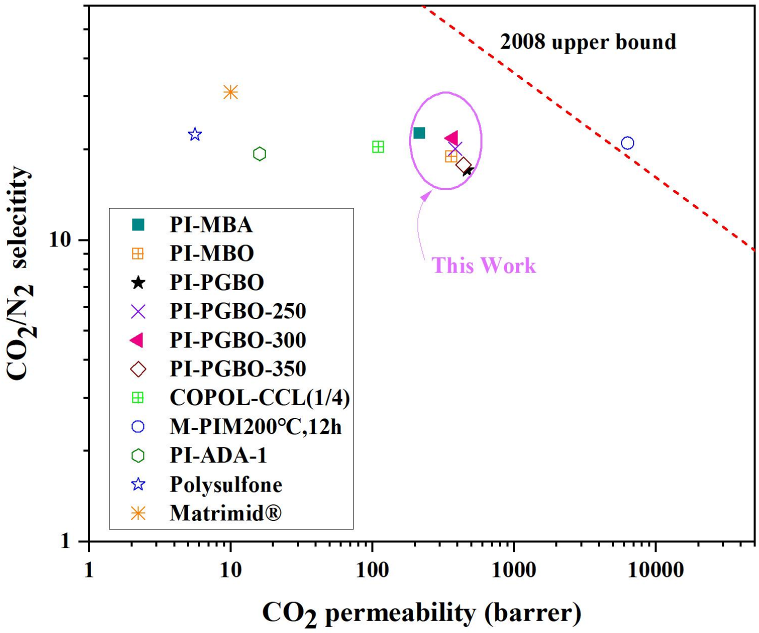

Fig. S2. Robeson Upper Bound Graph for CO_2/N_2 of Various Membranes [3-5].

4. Data on plasticization resistance of PI-PGBO and PI-PGBO-300

Table S1. Data on plasticization resistance of PI-PGBO and PI-PGBO-300.

**Polyimides**	**Feed pressure(bar)**	**CO_2 Permeability (Barrer)**
PI-PGBO	2.06	469.20
	5.16	398.94
	9.94	363.93
	15.1	373.87
	20.2	383.11
	25.27	438.22
	30.1	457.16
PI-PGBO-300	2.09	377.95
	5.2	350.12
	10.02	331.12
	15.38	320.95
	20.02	315.21
	24.89	311.46
	30.2	308.22

References

1. Kumazawa, H.; Wang, J.S.; Sada, E. Gas transport through homogeneous and asymmetric polyethersulfone membranes. *Journal of Polymer Science Part B: Polymer Physics* **2003**, *31*, 881-886, doi:10.1002/polb.1993.090310716.
2. Kanehashi, S.; Kusakabe, A.; Sato, S.; Nagai, K. Analysis of permeability; solubility and diffusivity of carbon dioxide; oxygen; and nitrogen in crystalline and liquid crystalline polymers. *Journal of Membrane Science* **2010**, *365*, 40-51, doi:10.1016/j.memsci.2010.08.035.
3. Robeson, L.M. The upper bound revisited. *Journal of Membrane Science* **2008**, *320*, 390-400, doi:10.1016/j.memsci.2008.04.030.
4. Zhu, Y.; Shen, J.; Yin, L.; Wei, X.; Chen, F.; Zhong, M.; Gu, Z.; Xie, Y.; Jin, W.; Liu, Z.; et al. Directly photopatterning of polycaprolactone-derived photocured resin by UV-initiated thiol-ene "click" reaction: Enhanced mechanical property and excellent biocompatibility. *Chemical Engineering Journal* **2019**, *366*, 112-122, doi:10.1016/j.cej.2019.02.045.
5. Eray, E.; Candelario, V.M.; Boffa, V. Ceramic Processing of Silicon Carbide Membranes with the Aid of Aluminum Nitrate Nonahydrate: Preparation, Characterization, and Performance. *Membranes (Basel)* **2021**, *11*, doi:10.3390/membranes11090714.
